# Measurement of melatonin in alcoholic and hot water extracts of *Tanacetum parthenium*, *Tripleurospermum disciforme* and *Viola odorata*

**Published:** 2010

**Authors:** M. Ansari, Kh. Rafiee, N. Yasa, S. Vardasbi, S.M. Naimi, A. Nowrouzi

**Affiliations:** 1 Department of Biochemistry, School of Medicine; 2 Department of Pharmacognosy, School of Pharmacy, Tehran University of Medical Sciences; 3 The Quality Control Department, Osveh Pharmaceutical Company, Tehran, Iran

**Keywords:** Melatonin, Medicinal herbs, HPLC, TLC, ELISA

## Abstract

**Background and the purpose of the study:**

Melatonin has recently been found in several plant tissues. Some reports show that the majority of the herbs containing the high level of melatonin have been used traditionally to treat neurological disorders or diseases associated with the generation of free radicals. Current study was undertaken to screen some medicinal plant species with historical evidence of efficacy in the treatment of neurological and antioxidant deficiency related disorders for their melatonin content. The melatonin content of boiled and alcoholic extracts were also compared.

**Methods:**

In this study, three medicinal herbs, *Tanacetum parthenium* (L.) Schultz. Bip. (Asteraceae), *Tripleurospermum disciforme* (C.A.Mey) Schultz. Bip. (Asteraceae) and *Viola odorata* (L.) (Violaceae) were analyzed using high performance liquid chromatography with ultraviolet detector (HPLC-UV), enzyme linked immunosorbent assay (ELISA) and thin layer chromatography (TLC).

**Results:**

Melatonin content in the dry plant powders differed with different assay methods (p < 0.001). For example, the melatonin content in *T. disciforme* was determined as 3.073 µg/g and 2.906 µg/g by the HPLC and the ELISA methods, respectively.

**Major conclusion:**

The results demonstrated that a hydroalcoholic solution could extract more melatonin from flowers of the herbs than hot water (p < 0.001). The presence of melatonin in these plant tissues may provide some explanation for the anecdotal evidence of their physiological effects in humans.

## INTRODUCTION

Melatonin is an indoleamine secreted by the pineal gland in mammals (1). This molecule has been linked to a number of the physiological and pathophysiological functions including regulation of circadian rhythms (1), prevention of ischemia - reperfusion damages (2), relief of chronic pain (3), enhancement of immunity (4), oncostatic effects (5), treatment of the neurological disorders such as migraine (6), antibacterial activity (7), anti-inflammatory, and antioxidative properties (8).

Several reports in the past decade have identified melatonin in different parts of plants and have opened up a new chapter in the field of plant- derived melatonin (phytomelatonin) (9). The majority of the herbs which contain high levels of melatonin have been used traditionally to treat neurological disorders (10) or diseases associated with the generation of free radicals (11) which could be due to the presence of this potent antioxidant molecule.

*Tanacetum parthenium* (L.) Schultz. Bip. (Asteraceae), *Tripleurospermum disciforme* (C.A. Mey) Schultz. Bip. (Asteraceae) and *Viola odorata* (L.) (Violaceae), which were evaluated in the present study, have a long history in the Iranian traditional medicine for the treatment of migraine (12), cancer (13) and menstrual cramps (12, 14), and also have been used as sedatives (15, 16), anti-microbial (17, 18) anti-inflammatory agents (12, 13, 15, 19), and other common problems related to stress (14, 18). Because of the known bioactivity of melatonin in different diseases, it was hypothesized that in addition to other active ingredients, the above plants might as well contain melatonin. Drinking the water extract or boiled plant parts is the most common method of using medicinal plants in household and in traditional medicine (20). Since no study has ever compared the melatonin content of boiled and the alcoholic extracts, the present study attempted to address these issues.

## MATERIAL AND METHODS

### 

#### Materials

*T. parthenium* (code: 84715) was collected from herboratum of Pharmacognosy Department of Tehran University of Medical Sciences; *T. disciforme* and *V. odorata* were purchased from a local market in Tehran, Iran and authenticated by Dr. Narges Yasa, Department of Pharmacognosy, Tehran University of Medical Sciences. Following shed drying of the plant materials, they were grounded in liquid nitrogen and stored at -70C° for a maximum of 2 weeks before extraction.

Melatonin and other chemicals were purchased from Sigma-Aldrich (St Louis, MO, USA). Organic solvents were from Merck (Darmstadt, Germany). Water was purified using an ultra clearing system (UV plus; SG, Barsbuettel, Germany).

#### Methods

##### Alcoholic Extracts

Herbal powders (10 g) were suspended in 50 mL of methanol: water (1:1) as the homogenizing solution (21) and ultrasonicated for 20 min at room temperature. The homogenates were centrifuged at 4000 g for 15 min (11). After removal of the supernatant, it was filtered (0.2 _ 25 mm, Millipore) and concentrated in glass vials under the nitrogen gas down to 10 ml.

##### Hot water Extracts

Herbal powder of the plant flowers (40g) were added to 1 liter of distilled water and extraction was performed by heating at 80°C (22). After the extract was filtered under suction through Whatman No. 1 filter paper (21), the crude extract was concentrated by a rotary evaporator (22) under reduced pressure at low temperature down to 40 ml. It was re-filtered by a filter (0.2 _ 25 mm, Millipore) prior to HPLC analysis.

Finally, in both of the extraction methods, the remaining extract was centrifuged and the supernatant was used for detection of melatonin by HPLC-UV.

##### HPLC

The HPLC system consisted of a reversed-phase Nucleosil-100 C18 column (Batch no. 21304102, 25cm × 4.6 mm ID, particle size 5 µm; Berlin, Germany) with integrated precolumn guard, an isocratic HPLC pump (2248 Pharmacia LKB, USA), and a Rheodyne injector with a 20µl sample loop. Melatonin retention time was determined using HPLC- Ultraviolet detector (VWM 2141; Pharmacia LKB, USA, wavelength at 280 nm). The mobile phase consisted of 0.1 M potassium phosphate buffer (pH 4.5) with acetonitrile (20%) at a flow rate of 1 ml/min.

**Figure 1:**
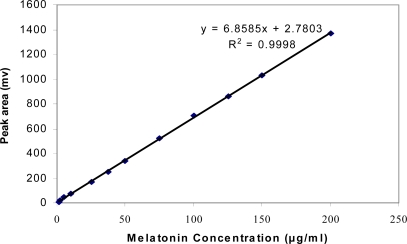
Relationship of the melatonin concentrations and the HPLC peak areas. The melatonin concentrations used to obtain this curve ranged from 0.5-200 µg/ml. The limit of detection (LOD) for melatonin was 0.5µg/mL.

Synthetic melatonin was dissolved in a mixture of water-methanol (1:1) and further dilutions were made immediately before the use. Calibration curve of melatonin was made using the HPLC-UV within the range of 0.5-200 µg/ml (Figure. 1). Melatonin concentrations were calculated on the basis of the HPLC peak areas. The limit of detection (LOD) for melatonin was 0.5µg/ml (2.2 nM) by this method.

##### HPLC purification

HPLC was used to purify plant extracts prior to ELISA (23) or TLC (24). A modified version of the methodology used by Pape et al. (23) was used in the present study. Briefly, the concentrated extracts were injected to the HPLC system. A 2-min fraction (2 ml) containing melatonin was collected and treated immediately with 2 ml of chloroform. After overnight incubation and centrifugation, the water phase was discarded and the organic phase transferred to a new glass tube.

For the TLC identification, the chloroform extracts of five runs of were concentrated to a small volume under nitrogen gas. For ELISA determinations, the chloroform of a single HPLC run was evaporated to dryness (23) and the residue was dissolved in de- ionized water and melatonin ELISA kit (25).

##### TLC

To verify that the HPLC-peak with a retention time of20 min was truly melatonin, the peak corresponding to melatonin on the chromatogram of the herb extract was identified by TLC on silica gel G TLC glass plates (5 × 20cm, Merck) with CHCl_3_ -MeOH (9:1). Eluate corresponding to the peak at retention time of 20 min was subjected to TLC along with authentic melatonin and the spots were visualized by UV irradiation (24). Spraying of the TLC plate by chromogenic Van Urk-Salkowski reagent was carried out in a fume hood, using a glass atomizer (26). Finally, the chromogenic spots were detected and their Rf values compared.

##### ELISA

Melatonin levels were quantified by an ELISA kit (EK-DSM; Buhlmann Laboratories AG, Schonenbuch, Switzerland) (23, 25) according to the manufacturer's instructions.

Briefly, the wells of the micro-titer plates, pre- coated with polyclonal Kennaway G280 anti- melatonin antibody, were filled in duplicate either with blank reagent, calibrators, and samples or self-made standard melatonin in concentration range of 0, 3, 10, 30, 100 and 300pg/ml. After an overnight incubation the procedure was followed by addition of other compounds of the kit. Finally, after addition of the stop solution (0.25 M sulfuric acid), the optical densities at 450 nm were measured by means of a micro-plate reader (Anthos 2020, Austria).

##### Statistical analyses

Data are expressed as means ±SD and determined using one-way analysis of variance (ANOVA). A *p* < 0.05 was considered to be statistically significant.

## RESULTS AND DISCUSSION

Melatonin was identified in extracts of herbs of this present study. The quality-control studies showed that the recovery of melatonin was approximately 80% which could be a means for calculation of the relative melatonin contents of the samples before homogenization and extraction with solvents. After correction of the values for the recovery rate, concentrations of melatonin were calculated in the flowers of the herbs by the HPLC-UV and ELISA methods (Table 1).

**Table 1:** Melatonin content in three medicinal herbs determined by HPLC-UV and ELISA methods.

Method	HPLC-UV Melatonin, ng/g±SD (d.w.)^a^ including recovery		ELISA Melatonin, ng/g±SD (d.w.)^a^ including recovery	
Plants				
	Hot water	Methanol 50%	Hot water	Methanol 50%
*Tanacetum parthenium*	1120.0 (±131.6)	2086.9 (±156.0)	1010.6 (±41.1)	1803.4 (±58.8)
*Triplourespermum disciforme*	1305.8 (±179.4)	3073.3 (±162.7)	1112.0 (±37.5)	2096.2 (±75.4)
*Viola odorata*	0.8 (±0.1)	Nd^b^	Nd^b^	1.1 (±0.1)

a Metabolite concentration expressed per gram of dried material.

b Melatonin was below detection limits.

**Figure 2:**
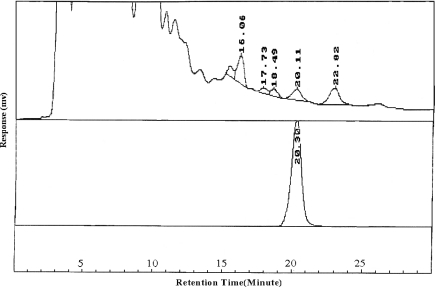
HPLC-UV peaks for T. parthenium extract in the upper panel (Rt= 20.1 min) compared to standard melatonin in the lower panel (Rt= 20.3 min) detected at a wavelength of 280 nm. The mobile phase consisted of 0.1 M potassium phosphate buffer (pH 4.5) with acetonitrile (20%) at a flow rate of 1 ml/min.

In flowers of *T. parthenium* high concentrations of melatonin were observed in all extraction methods; a result different from those of March et al (10) who found very high concentration of melatonin in the leaves of this plant but not in their flowers. The variables like intra-species differences, false-positive detection, destruction during extraction procedures (23), and environmental factors such as soil quality, temperature, and stress (27) affect melatonin levels in plants and may be reasons for this difference. To our knowledge, this may be the first time that *T. disciforme* has been checked for melatonin content. Values of melatonin in hot water and alcoholic extracts of this herb in comparison with other two plants were significantly higher by both ELISA and HPLC methods compared to other two plants (p<0.001) (Table 1). Previously, high melatonin levels have been reported in some medicinal plants such as Shiya tea-leaf, Chantui (11), and feverfew (10). It was not possible to detect a significant amount of melatonin in *V. odorata* flowers by HPLC, but a lower level was found in the same sample by ELISA (Table 1). HPLC chromatograms of the extract obtained from *T. parthenium* with synthetic melatonin are shown in figure 2.

The mean melatonin levels in the flowers of the herbs, obtained from the HPLC method, differed significantly from values obtained by ELISA (p<0.001) (Table 1). Since a single sample was used for both methods, this discrepancy might have resulted from differences in sensitivity and specificity of these methods; for example, in *V. odorata* it was possible to detect melatonin with ELISA method but not by HPLC method (sensitivity; HPLC=20ng, ELISA=0.2 pg). Otherwise, HPLC rather than ELISA method detected high levels of melatonin in *T. parthenium* and *T. disciforme* which might be due to difference in specificity of the methods. With respect to plant-derived indoleamines (28) and their possible coelution with melatonin (23), the HPLC method gave false-positive results. Interestingly, fractions obtained from HPLC and used in TLC method identified other components with different R_f_ values. (Not shown). Another reason for different values by HPLC and ELISA can be a possible destruction of melatonin in ELISA due to the length of time required the sample preparation.

Likewise, for the first time, TLC was applied for identification of phytomelatonin. In every herbal sample with TLC, the color of the indole condensation product was compared by eye with the standard that was bluish. The Rf value of the concentrate was 0.72 which was in agreement with that of synthetic melatonin (data not shown). TLC can create problems due to the presence of numerous indoleamine compounds in plant samples (28) which can be detected by Van Urk-Salkowski method (26). HPLC purification prior to TLC removed the above problems, and also confirmed that the sample obtained from HPLC was melatonin. However, in *V. odorata*, despite using several HPLC fractions for TLC, either because of low detection limit of TLC (50ng) and / or low melatonin level in this herb, it was not possible to identify melatonin. Such treatment of biologic samples for the purpose of TLC (24) or other kinds of analyses (23) has been reported previously. Results from this survey show that water fraction of the heated or boiled plant flowers contain considerable melatonin (Table 1). Although an appreciable amount of melatonin can be released from plants by hot water alone, the use of methanol could increase the efficiency of extraction (p<0.001) due to increase in solubility (21); the highest amount of melatonin was obtained using a 1:1 mixture of methanol and water. Of course, this difference could result from possible molecular destruction due to heat. It has been reported that the level of this indoleamine in non-heat-treated food products is much higher than the heated food products (25). For this reason, by calculating melatonin recovery it was tried to wave the problem.

Human studies have shown that vegetable consumption leads to urine excretion of 6-sulfatoxy melatonin (29); probably due to an increase plasma melatonin levels caused by these vegetables.Many studies confirm that the normal peak level of melatonin in human serum is 10-200 picogram/ml (1). Since the medicinal herbs of the present study contained melatonin in concentrations ranging from 0.8-3000 ng/g, it could be an excellent dietary source of melatonin for supplementing endogenously synthesized melatonin. Also, melatonin might be the molecule responsible for the pharmacological properties of these plants and the probable basis of their applications in traditional medicine.

## CONCLUSIONS

Hot water fraction of the flowers of the *T. parthenium*, *T. disciforme and V. odorata* contained considerable amounts of melatonin. However, the hydro-alcoholic solution extracted more melatonin from flowers of the herbs compared to hot water. The presence of melatonin in these plant tissues might provide some explanation for the anecdotal evidence of their physiological effects in humans. These herbs should be re-evaluated in reference to their nutritional and medicinal values and the possibility of their application in other disease conditions associated with melatonin.
